# Supplementary material for: Elevational surveys of Sulawesi herpetofauna 1: Gunung Galang, Gunung Dako Nature Reserve
Source: PeerJ. 2023 Aug 21;11:e15766. doi: 10.7717/peerj.15766 (PMC10448876; doi:10.7717/peerj.15766)
Supplement: Supplemental Information 1 [file peerj-11-15766-s001.pdf]

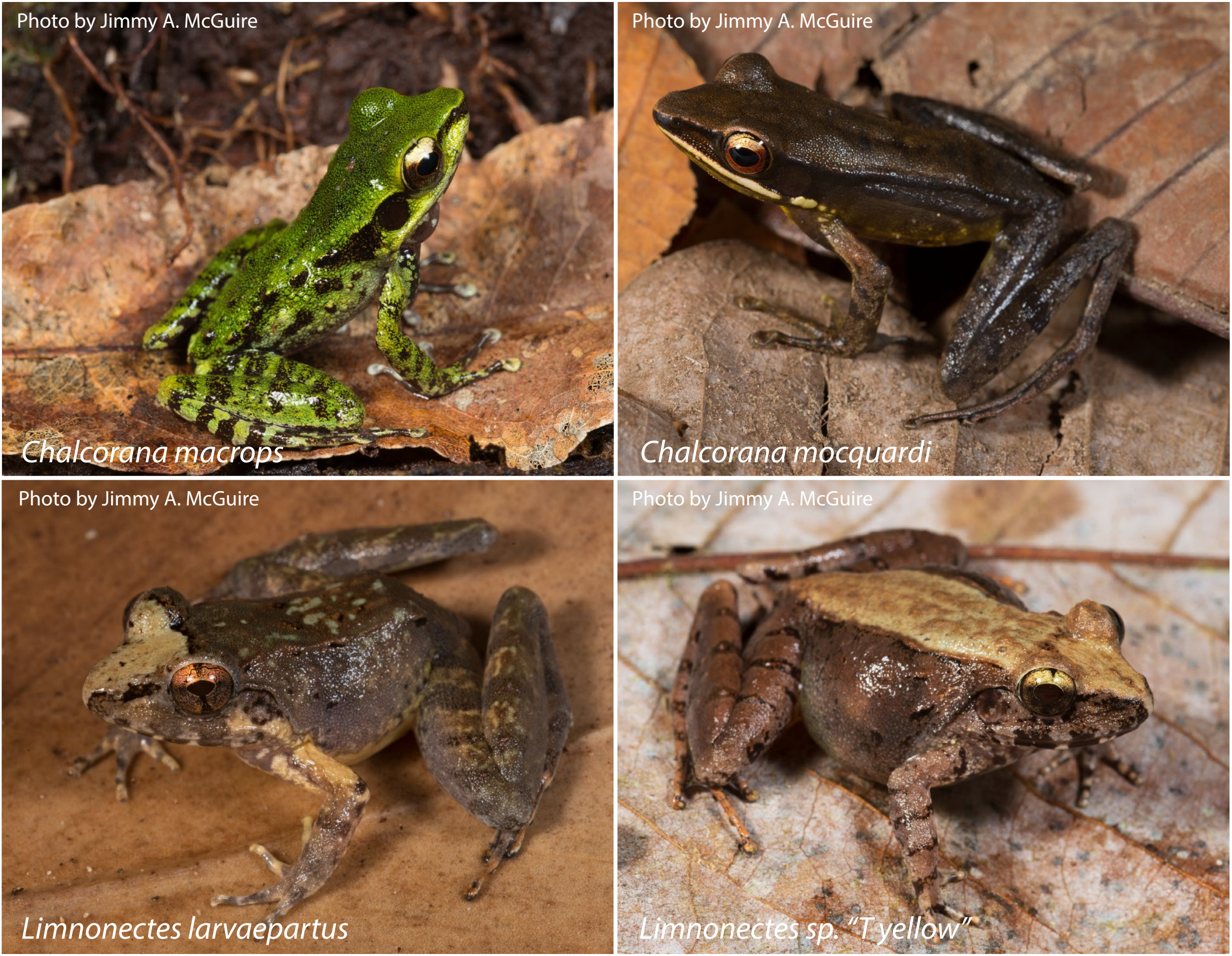
Photo by Jimmy A. McGuire
Chalcorana macrops
Photo by Jimmy A. McGuire
Chalcorana mocquardi
Photo by Jimmy A. McGuire
Limnonectes larvaepartus
Photo by Jimmy A. McGuire
Limnonectes sp. "T yellow"

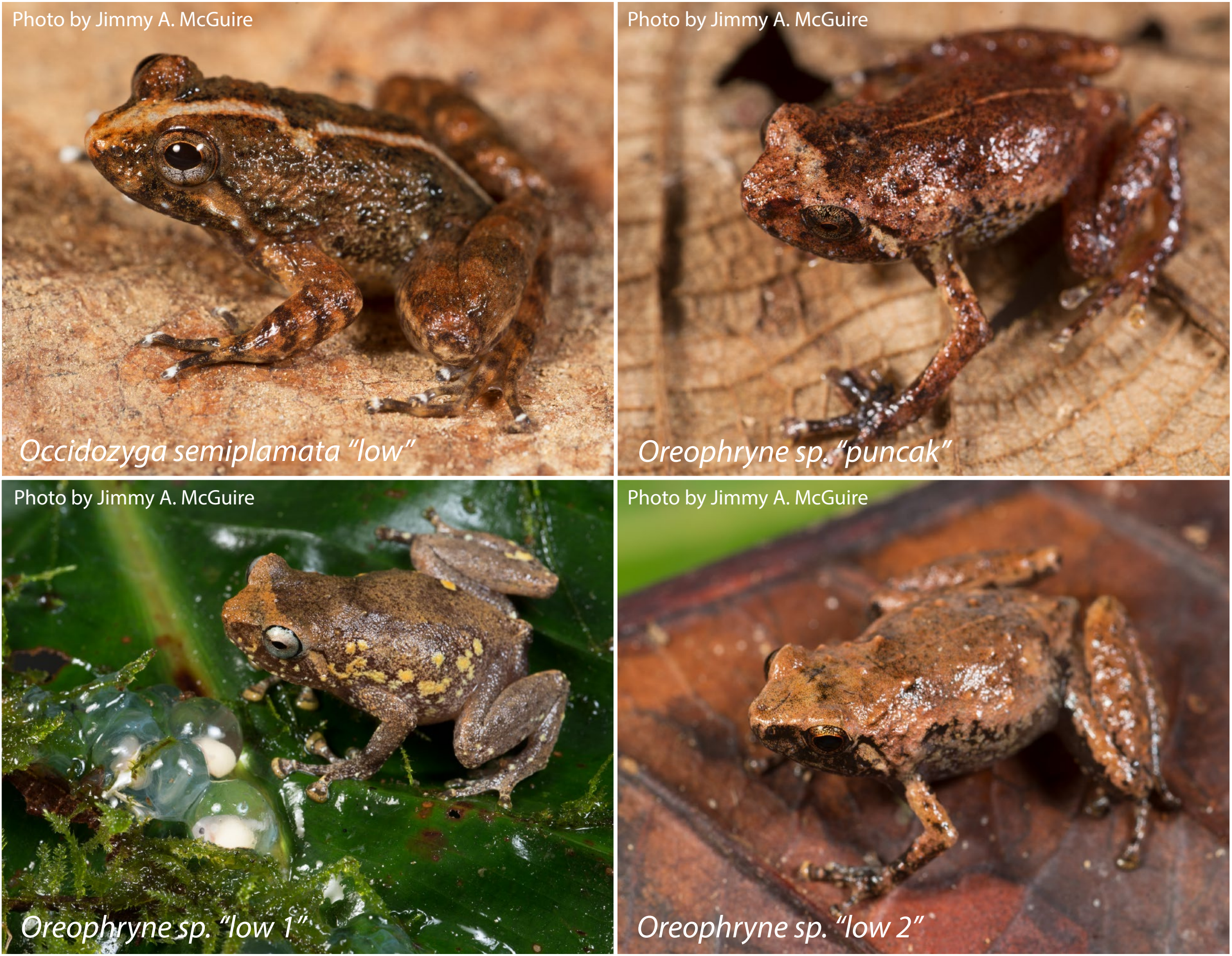
Photo by Jimmy A. McGuire
Occidozyga semiplamata "low"
Photo by Jimmy A. McGuire
Oreophryne sp. "puncak"
Photo by Jimmy A. McGuire
Oreophryne sp. "low 1"
Photo by Jimmy A. McGuire
Oreophryne sp. "low 2"

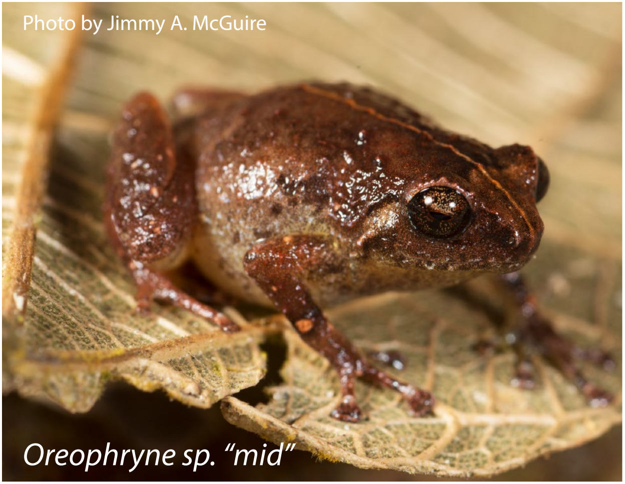
Photo by Jimmy A. McGuire
Oreophryne sp. "mid"

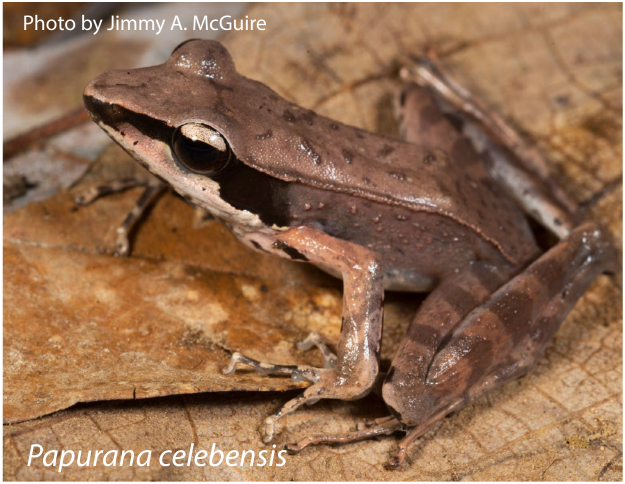
Photo by Jimmy A. McGuire
Papurana celebensis

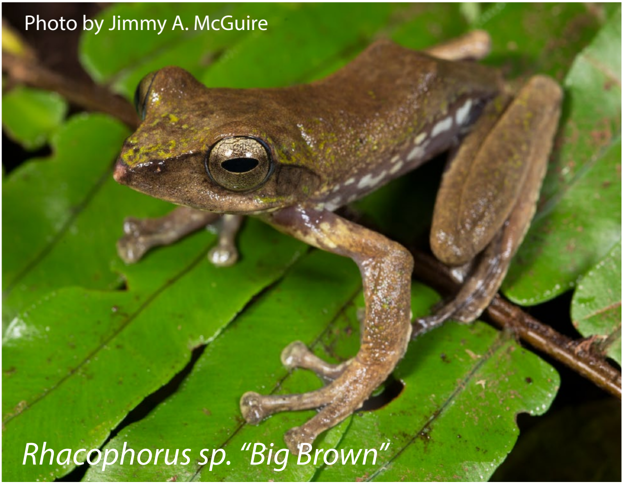
Photo by Jimmy A. McGuire
Rhacophorus sp. "Big Brown"

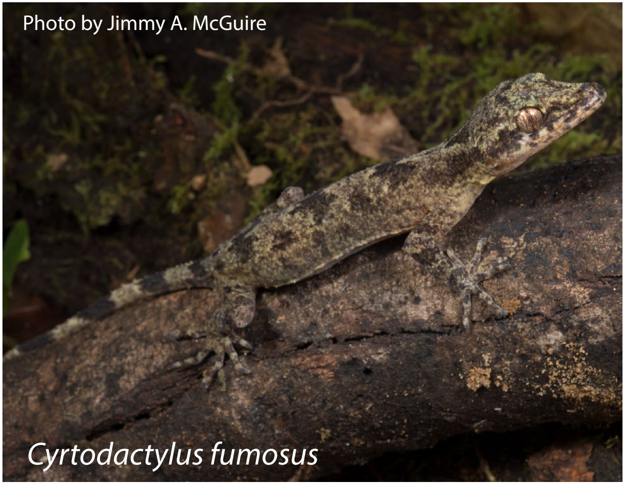
Photo by Jimmy A. McGuire
Cyrtodactylus fumosus

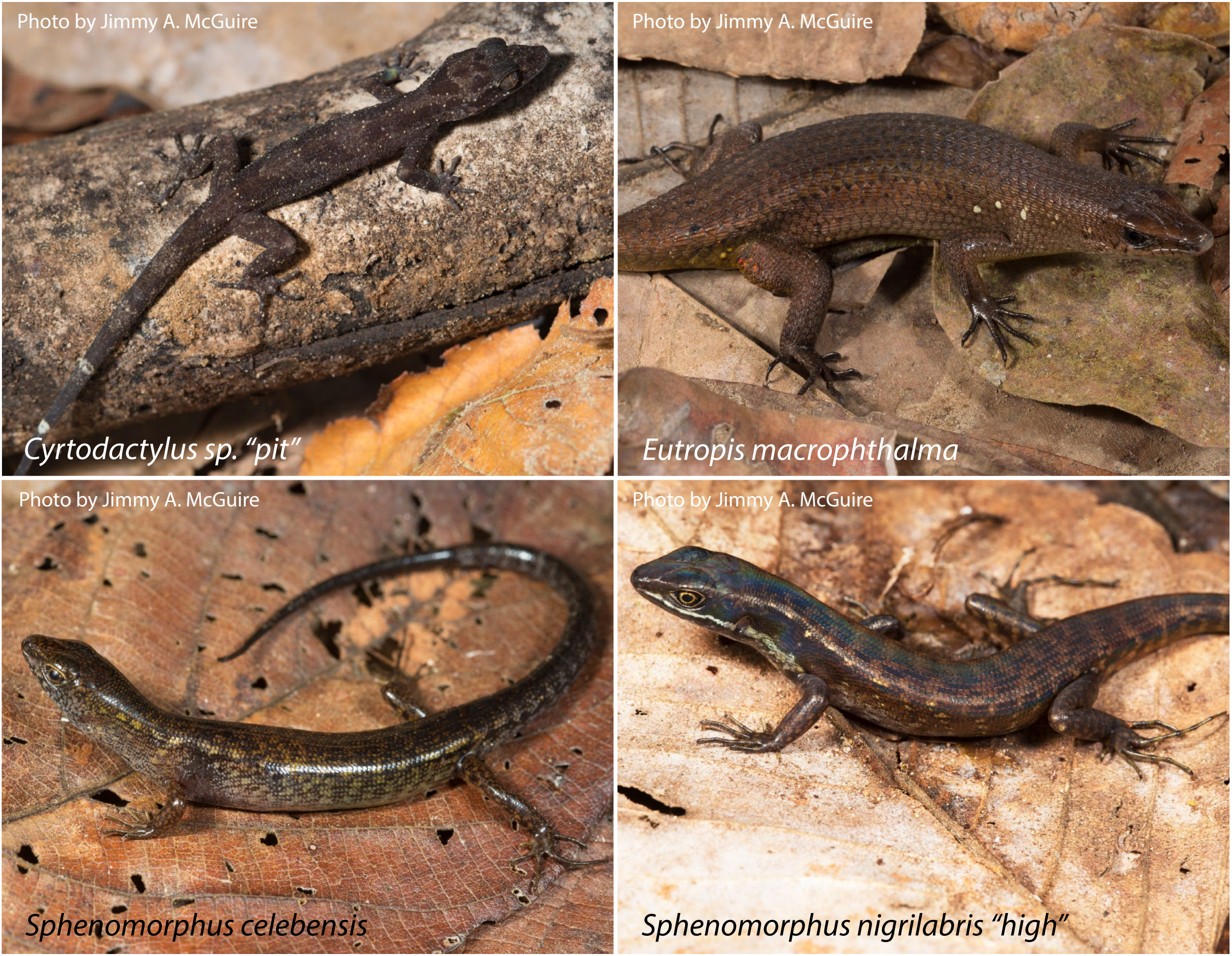
Photo by Jimmy A. McGuire
Cyrtodactylus sp. "pit"
Photo by Jimmy A. McGuire
Eutropis macrophthalma
Photo by Jimmy A. McGuire
Sphenomorphus celebensis
Photo by Jimmy A. McGuire
Sphenomorphus nigrilabris "high"

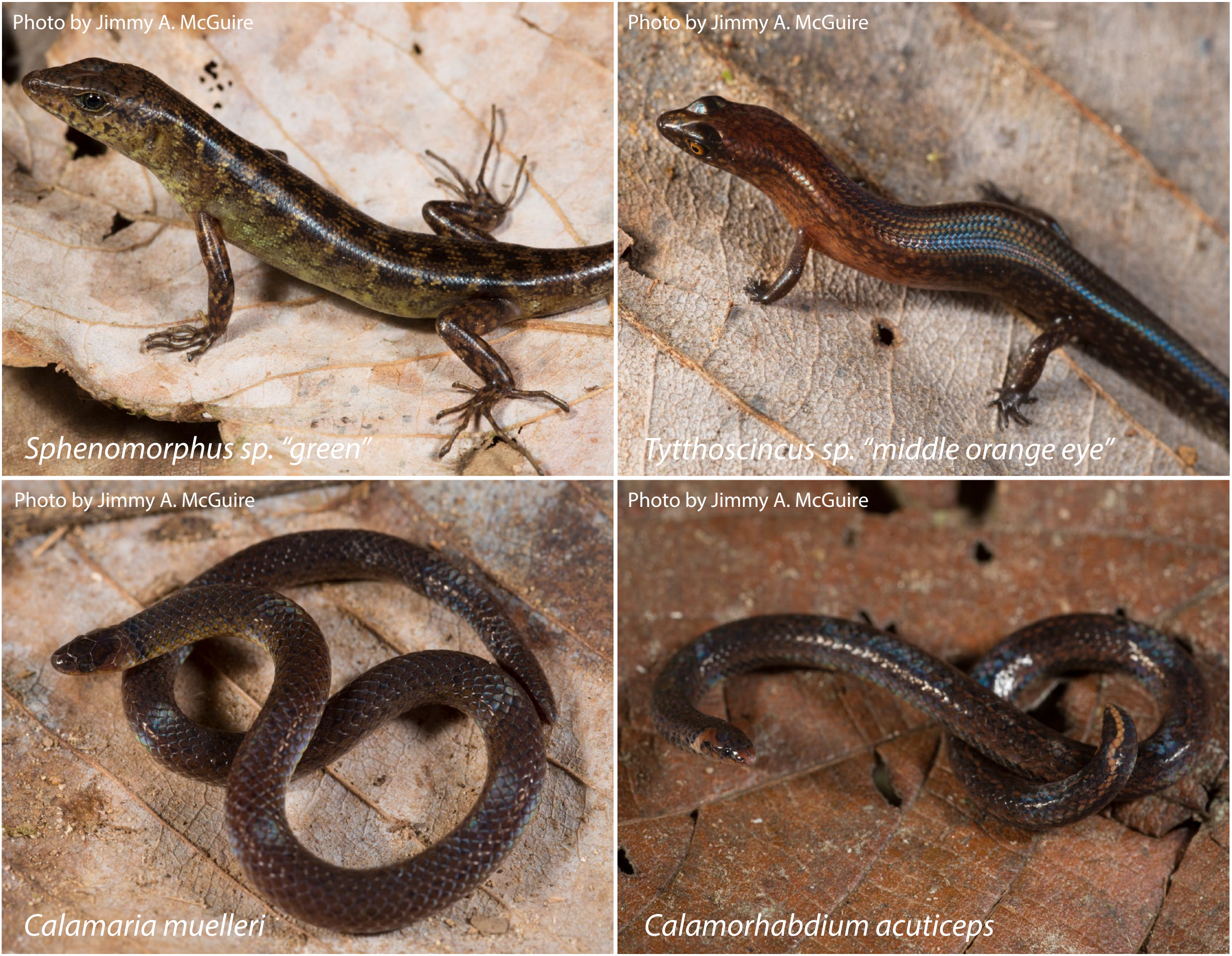
Photo by Jimmy A. McGuire
Sphenomorphus sp. "green"
Photo by Jimmy A. McGuire
Tytthoscincus sp. "middle orange eye"
Photo by Jimmy A. McGuire
Calamaria muelleri
Photo by Jimmy A. McGuire
Calamorhabdium acuticeps

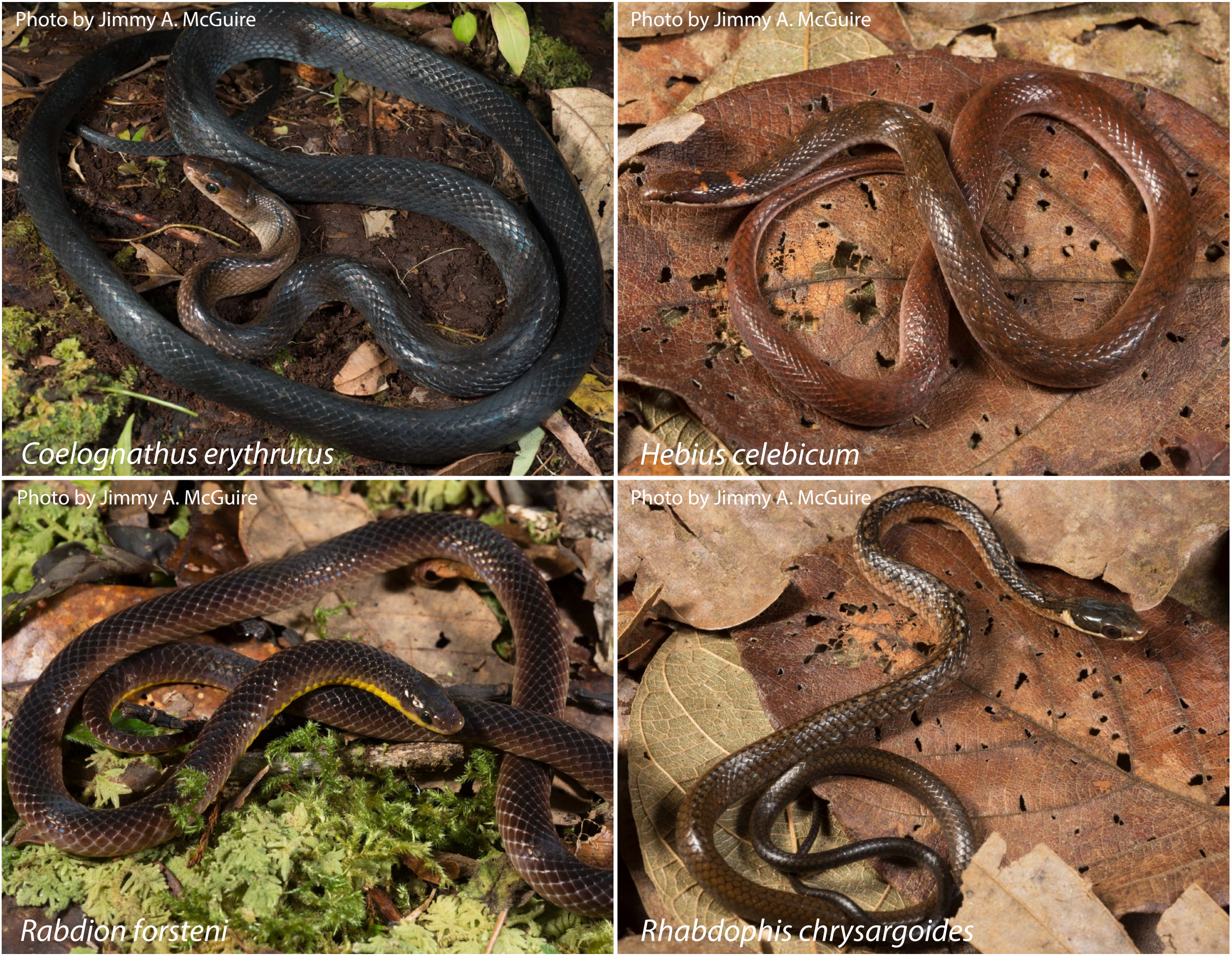
Photo by Jimmy A. McGuire
Coelognathus erythrurus
Photo by Jimmy A. McGuire
Hebius celebicum
Photo by Jimmy A. McGuire
Rabdion forsteni
Photo by Jimmy A. McGuire
Rhabdophis chrysargoides

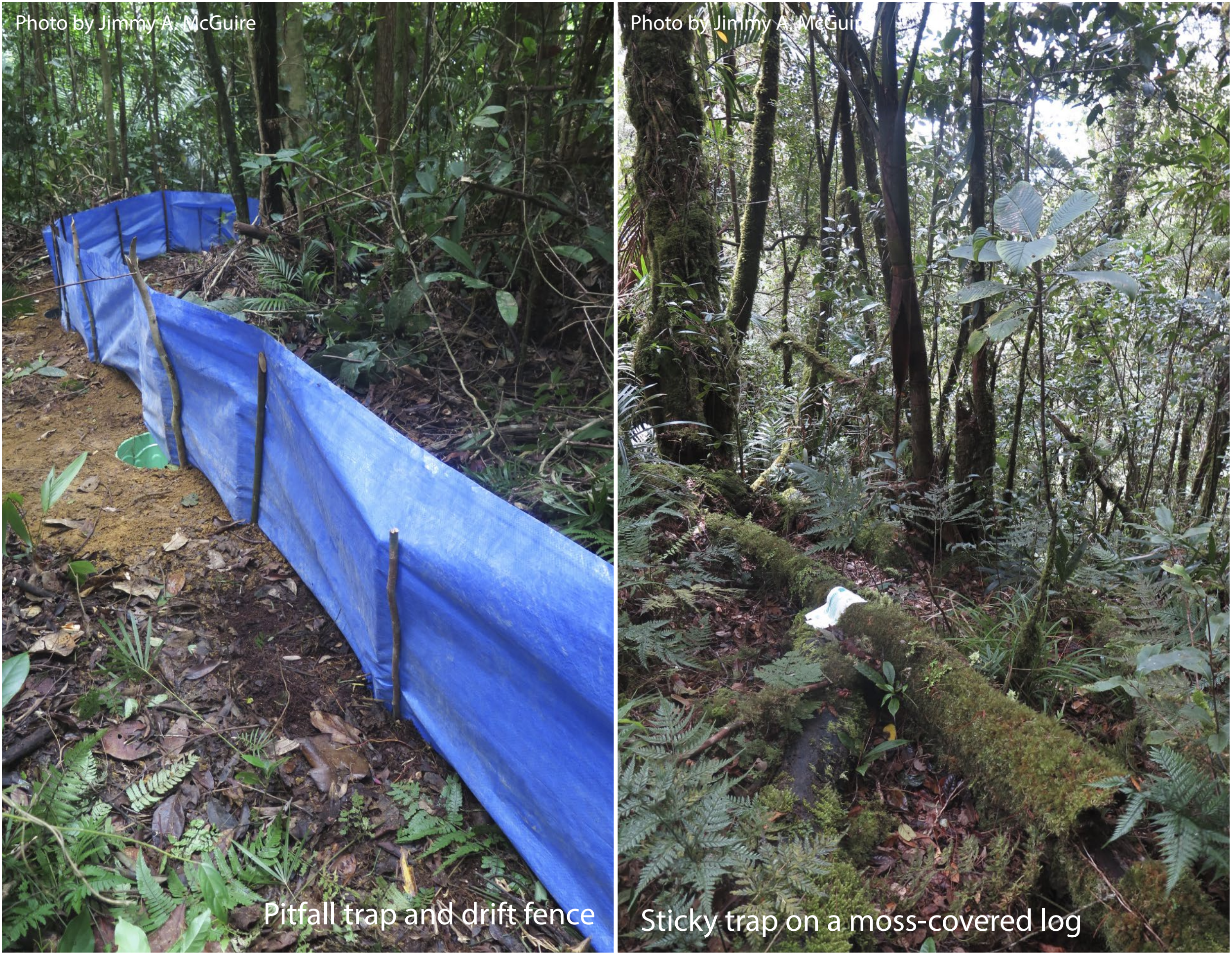
Photo by Jimmy A. McGuire
Pitfall trap and drift fence
Photo by Jimmy A. McGuire
Sticky trap on a moss-covered log
