# Supplementary material for: Elevational surveys of Sulawesi herpetofauna 1: Gunung Galang, Gunung Dako Nature Reserve
Source: PeerJ. 2023 Aug 21;11:e15766. doi: 10.7717/peerj.15766 (PMC10448876; doi:10.7717/peerj.15766)
Supplement: Supplemental Information 2 [file peerj-11-15766-s002.docx]

**Abstract — Bahasa Indonesia**

Pulau Sulawesi, Indonesia memiliki sejarah geologi dan geografi yang unik. Karenanya pulau ini memiliki keragaman spesies flora dan fauna serta spesies endemik yang menakjubkan. Lebih dari itu, spesies flora fauna tersebut juga memiliki sejarah biogeografi yang sangat menarik. Hingga saat ini, sebagian besar penelitian mengenai keragaman hayati Sulawesi hanya berfokus pada jenis endemik yang berada di bagian tengah (Central Core) and di empat semenanjung pulau ini. Studi keragaman spesies yang ada di dataran tinggi pulau ini masih sangat terbatas, termasuk untuk amfibi dan reptil. Ini adalah paper pertama dari rangkaian paper yang kami siapkan untuk menyajikan daftar spesies herpetofauna dari gunung-gunung di Sulawesi berdasarkan hasil expedisi yang kami lakukan. Dalam jangka waktu survey selama tiga minggu di Gunung Galang (2245 m) yang terletak di bagian barat kota Tolitoli, Provinsi Sulawesi Tengah, di semenanjung utara pulau Sulawesi, kami mencatat sekitar 50 spesies amfibi dan reptil. Sekitar 10 spesies diantaranya kemungkinan adalah spesies baru atau spesies yang sudah diketahui namun belum dideskripsi. Sampling kami yang tidak menyeluruh di Gunung Galang menunjukan bahwa masih banyak lagi spesies yang belum ditemukan, baik di gunung ini maupun di daerah sekitarnya.
